# Effects of Oral L-Carnitine Administration in Narcolepsy Patients: A Randomized, Double-Blind, Cross-Over and Placebo-Controlled Trial

**DOI:** 10.1371/journal.pone.0053707

**Published:** 2013-01-17

**Authors:** Taku Miyagawa, Hiromi Kawamura, Mariko Obuchi, Asuka Ikesaki, Akiko Ozaki, Katsushi Tokunaga, Yuichi Inoue, Makoto Honda

**Affiliations:** 1 Department of Human Genetics, Graduate School of Medicine, The University of Tokyo, Tokyo, Japan; 2 Clinical Trial Operations Division, Site Support Institute Co., Ltd. (SSI), Tokyo, Japan; 3 School of Nursing, Faculty of Nursing, Toho University, Tokyo, Japan; 4 Japan Somnology Center, Neuropsychiatric Research Institute, Tokyo, Japan; 5 Department of Somnology, Tokyo Medical University, Tokyo, Japan; 6 Sleep Disorders Project, Department of Psychiatry and Behavioral Sciences, Tokyo Metropolitan Institute of Medical Science, Tokyo, Japan; Catholic University of Sacred Heart of Rome, Italy

## Abstract

Narcolepsy is a sleep disorder characterized by excessive daytime sleepiness, cataplexy, and rapid eye movement (REM) sleep abnormalities. A genome-wide association study (GWAS) identified a novel narcolepsy-related single nucleotide polymorphism (SNP), which is located adjacent to the carnitine palmitoyltransferase 1B (*CPT1B*) gene encoding an enzyme involved in β-oxidation of long-chain fatty acids. The mRNA expression levels of *CPT1B* were associated with this SNP. In addition, we recently reported that acylcarnitine levels were abnormally low in narcolepsy patients. To assess the efficacy of oral l-carnitine for the treatment of narcolepsy, we performed a clinical trial administering l-carnitine (510 mg/day) to patients with the disease. The study design was a randomized, double-blind, cross-over and placebo-controlled trial. Thirty narcolepsy patients were enrolled in our study. Two patients were withdrawn and 28 patients were included in the statistical analysis (15 males and 13 females, all with HLA-DQB1*06:02). l-carnitine treatment significantly improved the total time for dozing off during the daytime, calculated from the sleep logs, compared with that of placebo-treated periods. l-carnitine efficiently increased serum acylcarnitine levels, and reduced serum triglycerides concentration. Differences in the Japanese version of the Epworth Sleepiness Scale (ESS) and the Medical Outcomes Study 36-Item Short-Form Health Survey (SF-36) vitality and mental health subscales did not reach statistical significance between l-carnitine and placebo. This study suggests that oral l-carnitine can be effective in reducing excessive daytime sleepiness in narcolepsy patients.

**Trial Registration:**

University hospital Medical Information Network (UMIN) UMIN000003760

## Introduction

Narcolepsy is a sleep disorder characterized by excessive daytime sleepiness, cataplexy (sudden loss of muscle tone in response to strong emotions), and rapid eye movement (REM) sleep abnormalities. Narcolepsy patients are at a higher risk of obesity and non–insulin-dependent diabetes mellitus [1]–[5], and show high levels of total cholesterol and triglycerides [6]. The prevalence of narcolepsy is 0.16% to 0.18% in Japan and 0.02% to 0.06% in the United States and Europe [7]. Following studies in dogs [8] and mice [9], a 95% loss of orexin (hypocretin)-producing cells in postmortem hypothalami from narcolepsy patients was reported [10], [11]. It has been reported that both genetic and environmental factors contribute to its development [7]. Narcolepsy is closely associated with human leukocyte antigen (*HLA*)-DQB1*06:02 [12], [13]. Results from a genome-wide association study (GWAS) have revealed significant associations between narcolepsy and single nucleotide polymorphisms (SNP) located in the T-cell receptor α-locus and *P2RY11* [14], [15]. Recently, we also conducted a GWAS and identified a novel narcolepsy-related SNP (rs5770917), located adjacent to the gene encoding carnitine palmitoyltransferase 1B (CPT1B) [16]. The mRNA expression levels of *CPT1B* were associated with this SNP and the expression levels were decreased according to the number of risk alleles (C) [16], [17].

CPT1B is a rate-limiting enzyme in β-oxidation of long-chain fatty acids, which is mainly localized in the muscle mitochondrial outer membrane [18]. Conjugation of carnitine to long-chain fatty acyl coenzyme A (CoA) by CPT1B allows the transport of long-chain fatty acids into the mitochondrial matrix for subsequent β-oxidation. Several reports have indicated a role for fatty acid β-oxidation and the carnitine system in sleep regulation. Fasted juvenile visceral steatosis (jvs^−/−^) mice with systemic carnitine deficiency exhibit a higher frequency of fragmented wakefulness and REM sleep, and reduced locomotor activity [19]. These phenotypes in the fasted jvs^−/−^ mice are similar to those in mouse models of narcolepsy [9], [20]. In these mice, a significant reduction in the number of c-Fos-positive orexin neurons, hypothalamic prepro-orexin mRNA expression, and orexin-A concentration in the cerebrospinal fluid (CSF) was observed [19], [21]. These findings indicate that the acylcarnitine availability is essential for normal sleep regulation and orexin cell functions. On the other hand, mice deficient in short-chain acyl-CoA dehydrogenase (encoded by *Acads*), an enzyme catalyzing the first step of β-oxidation, have shown significantly slower theta frequency during REM sleep [22]. Administration of acetyl-l-carnitine, which is known to restore β-oxidation in the mitochondria [23], [24], significantly recovers slow theta frequency in the mutant mice.

In a previous study, we analyzed the expression level of *CPT1B* and measured the carnitine fractions in blood samples obtained from narcolepsy patients and healthy control subjects [17]. *CPT1B* expression was significantly higher in the narcolepsy patients than in the controls, and acylcarnitine levels were abnormally low in 21% of the narcolepsy patients while those of all the controls were within the normal range, suggesting that fatty acid β-oxidation is altered in narcolepsy [17]. Therefore, we hypothesized that promoting fatty acid oxidation by l-carnitine supplementation could alleviate narcolepsy symptoms.

## Materials and Methods

### Patients

Suitable study patients were identified from consecutive patients attending the Yoyogi sleep clinic affiliated to Neuropsychiatric Research Institute. Inclusion criteria were: age≥15 years and patients satisfying the diagnostic criteria of the 2nd edition of the International Classification of Sleep Disorders (ICSD-2) for narcolepsy with cataplexy. Written informed consent was obtained from all study participants. We did not obtain informed written consent from a legally acceptable representative because the participants of this study were all at least 20 years old and competent to consent. Thirty narcolepsy patients were enrolled in our study. Two patients were dropped out due to inability to follow regular visits defined in the study protocol and 28 patients were included in the statistical analysis (15 males and 13 females). The mean age ± standard deviation (SD) was 41.2±15.9 years. All the patients carried the HLA-DQB1*06:02 and exhibited unambiguous cataplexy. Exclusion criteria were: pregnancy, potentially pregnant or lactating women; known hypersensitivity to l-carnitine; epilepsia; use of acenocoumarol or other experimental treatment during this study. The patients were unrelated Japanese individuals living in Tokyo or in neighboring areas. The protocol for this trial and supporting CONSORT checklist are available as supporting information; see Checklist S1 and Protocol S1. This study was approved by the Ethics Committee of the Tokyo Metropolitan Institute of Medical Science and the Ethics Committee of the Neuropsychiatric Research Institute. The gene analysis in this study was also approved by Human Genome, Gene Analysis Research Ethics Committee of the Faculty of Medicine and Graduate School of Medicine of the University of Tokyo.

### Design

The trial was a randomized, double-blind, cross-over and placebo-controlled design of 16 weeks' duration. There were two, 8-week treatment periods, treatment period one and treatment period two. There were five specified visits at 0, 4, 8, 12 and 16 weeks. Patients were randomly assigned using a random number to l-carnitine during treatment period one, followed by placebo in treatment period two (group A), or placebo in treatment period one followed by l-carnitine in treatment period two (group B). An 8-week supply of the relevant treatment (l-carnitine or placebo capsules in a bottle) was labeled with that particular number. The randomization and labeling of the treatments were performed by a person who had not seen the patients. Oral l-carnitine (Otsuka Pharmaceutical Co., Ltd., Tokyo, Japan) and placebo were supplied in identical capsules. Dosage was three l-carnitine capsules (170 mg×3 = 510 mg) or three placebo capsules per day, two capsules in the morning and one capsule in the evening. l-carnitine crosses the BBB through carnitine transporter OCTN2 [25]. Concomitant medication for narcolepsy such as psychostimulants (modafinil, methylphenidate, pemoline for excessive daytime sleepiness and clomipramine for cataplexy and hypnagogic hallucination) was available on the condition that the dosage and administration of the medication would not be altered during this study. This study was registered at the University hospital Medical Information Network (UMIN) (ID: UMIN000003760).

### Efficacy variables and statistics

The primary outcome measure was the patient's subjective assessment of their sleepiness using total time for dozing off during the daytime in their sleep logs. The secondary outcome measures were as follows: the number of occurrences of dozing off during daytime, cataplexy and sleep paralysis in sleep logs; the Japanese version of the Epworth Sleepiness Scale (JESS) [26]; the Medical Outcomes Study 36-Item Short-Form Health Survey (SF-36) vitality (VT) and mental health (MH) subscales [27], [28]; Body Mass Index (BMI). Scores for JESS range from 0 to 24, with lower scores indicating less daytime sleepiness. Scores for SF-36 were transformed to norm-based scores with a Japanese population mean of 50 and a standard deviation (SD) of 10, with higher scores indicating a better health state. Actigraph data (Actiwatch, Phillips-Respironics, Tokyo) was collected for 11 days in both l-carnitine and placebo periods as auxiliary data to check the accuracy of sleep logs. We also evaluated the following biochemical measurements: serum levels of total carnitine, free carnitine, acylcarnitine, total cholesterol and triglycerides, measured by SRL Inc. (Tokyo, Japan). We did not adopt sleep log data for 2 weeks after the beginning of each treatment period in order to avoid a carry-over effect (treatment-period interaction). The total time and the number of times dozing off during daytime did not include scheduled daytime naps. Continuous variables were summarized as the mean and SD. Treatment effects, period effects and treatment-period interaction were analyzed using a 2-sample t-test. For the treatment effects, we used a one-tailed test because l-carnitine treatment was expected to improve narcolepsy symptoms relative to placebo controls. Baseline comparisons were performed with a 2-sample t-test. Categorical variables were summarized as percentages and the differences between groups were assessed using a chi-squared test. When data did not follow a normal distribution, non-parametric tests such as the Mann-Whitney U-test or the Wilcoxon signed-rank test were applied. Genotyping for SNP rs5770917 was performed using Taqman SNP genotyping assays (Life Technologies, Carlsbad, CA, USA) according to the manufacturer's protocol. IBM SPSS Statistics 19 and Microsoft Office Excel 2007 were used for statistical analyses. Baseline SF-36 scores from three subjects were missing due to incomplete fill-in. We conducted a power analysis using G*Power [29], [30] to determine the minimum sample size needed to detect a difference of 12 minutes in total time for dozing off during daytime. A total of 27 participants were required to achieve a power of 0.8 at a = 0.05 (one-tailed test). Clinical research coordinators sent from Site Support Institute Co., Ltd. (SSI) supported doctors to improve the quality of this study implementation.

## Results

**Figure 1:**
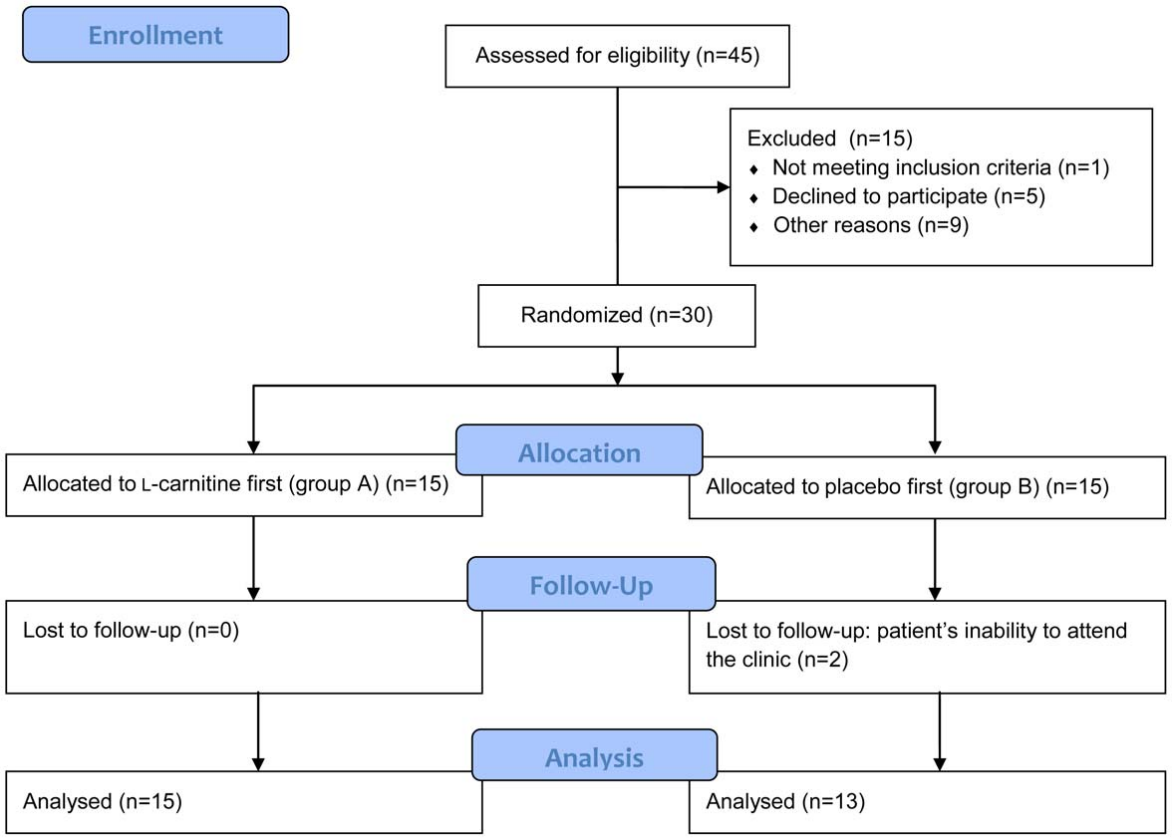
Study flow diagram.

A total of 30 narcolepsy patients were enrolled in this study between May, 2010 and July, 2010. Of the 30 enrolled patients, 28 completed all five visits defined in the study protocol (Fig. 1). There were no significant differences in the baseline characteristics of the study participants between the two groups (Table 1). The average BMI (± SD) of the 28 patients was 26.1±5.7, and 14 out of the 28 patients were obese. The average compliance (± SD) based on the number of capsules at clinic visits and patient logs was 98.2±2.7% on l-carnitine and 97.7±3.9% on placebo.

**Table 1:** Baseline characteristics.

Demographics	l-carnitine First	Placebo First	*P* Value
	(group A)	(group B)	
Male (%)/Female (%)	8(53%)/7 (47%)	7 (54%)/6 (46%)	0.97
Age at start of trial (SD)	41 (17)	42 (16)	0.88
BMI (SD)	24.9 (3.9)	27.5 (7.2)	0.24
Patients with BMI≥25 (%)	7 (47%)	7 (54%)	0.70
JESS (SD)	13.7 (2.9)	13.8 (4.0)	0.93
SF-36 VT (SD)	45.9 (11.3)	44.9 (12.1)	0.82
SF-36 MH (SD)	51.4 (8.3)	49.4 (7.9)	0.53

In Japan, obesity is diagnosed as a BMI≥25 according to the classification of obesity developed by the Japan Society for the Study of Obesity.

VT, Vitality Mental health; MH, Mental health.

Efficacy results are shown in Table 2. Regarding the primary endpoint, patients treated with l-carnitine showed a significant reduction in the total time for dozing off during daytime as measured by sleep logs, compared with the placebo period (l-carnitine: 49±34 min/day slept; placebo: 58±37 min/day; *P* = 0.048). The number of naps in patients given l-carnitine was also decreased, but not significantly (*P* = 0.14). There were no significant improvements in JESS and SF-36 subscale (vitality and mental health) scores between l-carnitine and placebo periods. However, the JESS scores of both periods and SF-36 subscale scores for the l-carnitine period tended to be improved from the baseline. The average numbers of episodes of cataplexy and sleep paralysis were low with less than 0.05 per day in both periods and showed no significant differences between treatments. Regarding BMI, no significant differences between l-carnitine and placebo periods were found. No period effects and no treatment-period interactions were observed for any of the parameters described in Table 2. l-carnitine was well-tolerated with no side effects observed. Out of 28 patients, 13 patients carried a risk allele of SNP rs5770917 (2 of them were homozygous for the risk allele) and 15 patients did not carry it. We tested whether SNP rs5770917 affects the primary endpoint (total time for dozing off during daytime). SNP rs5770917 was not significantly associated with the change of total dozing off time between l-carnitine and placebo periods (mean change [±SD] of risk allele carrier and non-carrier, 11.5±28.9 vs 6.4±26.2 minutes shortening; *P* = 0.62 [2-sample t-test]).

**Table 2:** Treatment effects of l-carnitine administration in narcolepsy patients.

Variable	l-carnitine	Placebo	*P* Value
Total nap time/day, min (SD)	49 (34)	58 (37)	0.048
No. of naps/day (SD)	1.3 (1.2)	1.4 (1.0)	0.14
No. of cataplexy/day (SD)	0.04 (0.07)	0.02 (0.04)	0.52*
No. of sleep paralysis/day (SD)	0.02 (0.06)	0.01 (0.03)	0.66*
JESS (SD)	12.7 (3.8)	12.7 (3.7)	0.48
difference to baseline (SD)	−1.0 (2.2)	−1.0 (3.8)	
SF-36 VT (SD)	47.5 (10.3)	45.5 (10.7)	0.13
difference to baseline (SD)	3.3 (7.1)	0.3 (10.0)	
SF-36 MH (SD)	50.4 (7.4)	49.9 (7.7)	0.39
difference to baseline (SD)	1.4 (8.2)	−0.2 (9.6)	
BMI (SD)	26.0 (5.8)	26.1 (5.9)	0.19
difference to baseline (SD)	−0.1 (0.6)	0.1 (0.7)	

* Parametric test assumptions were not available; thus the comparisons were performed with the Mann-Whitney U-test. For comparisons which were not analyzed using the Mann-Whitney U-test, the *P* values were calculated with a 2 sample t-test.

VT, Vitality Mental health; MH, Mental health.

Underlined variable, total nap time, indicates the primary endpoint of this study.

Table 3 shows the effects of l-carnitine on metabolic characteristics. Narcolepsy patients with placebo showed relatively low levels of acylcarnitine (9.4±3.3 µmol/L; normal range: 6–23 µmol/L; 4 patients abnormally low), as we found abnormally low acylcarnitine levels in narcolepsy patients in the previous study [17]. The average level of acylcarnitine was significantly increased to 12.8±4.6 µmol/L (*P* = 3.1×10^−5^), and 3 out of the 4 patients reached the normal levels by the administration of l-carnitine. Total and free carnitine levels were elevated by the treatment as we expected. Triglyceride levels during the l-carnitine treatment period were significantly decreased compared with those during the placebo period (*P* = 0.028), and the average level dropped to the normal level (l-carnitine: 132.9±79.5 mg/dL; placebo: 168.7±111.4 mg/dL; normal range: 50–149 mg/dL). Four of the 28 patients were treated for hyperlipidemia. Triglyceride levels in the remaining 24 patients were compared between l-carnitine and placebo periods in order to eliminate the influence of the treatment for hyperlipidemia. A significant reduction for triglyceride levels was also observed in l-carnitine period (mean [±SD], l-carnitine: 122.8±81.1 mg/dL; placebo: 168.1±118.9 mg/dL; *P* = 0.014). There was no significant difference for total cholesterol. No period effects and no treatment-period interactions were found for any of the parameters described in Table 3.

**Table 3:** Metabolic characteristics in l-carnitine and placebo periods.

Variable	l-carnitine	Placebo	*P* Value
Acylcarnitine (SD), µmol/L	12.8 (4.6)	9.4 (3.3)	3.1×10^−5^
Total carnitine (SD), µmol/L	66.6 (10.7)	54.1 (9.4)	3.5×10^−9^
Free carnitine (SD), µmol/L	53.8 (8.5)	44.6 (7.5)	1.2×10^−8^
Triglycerides (SD), mg/dL	132.9 (79.5)	168.7 (111.4)	0.028
Total cholesterol (SD), mg/dL	213.8 (44.7)	206.3 (42.9)	0.96

The normal ranges for the laboratory tests are as follows: acylcarnitine 6–23 µmol/L; total carnitine 45–91 µmol/L, free carnitine 36–74 µmol/L, triglycerides 50–149 mg/dL and total cholesterol 150–219 mg/dL.

## Discussion

The present study revealed that total time for dozing off during daytime in narcolepsy patients, the primary endpoint, was significantly decreased by l-carnitine administration compared with placebo. Secondary outcome measures showed no statistically significant improvement between l-carnitine and placebo periods. However, the number of episodes of dozing off during daytime, JESS and SF-36 scores during the l-carnitine treatment period showed a tendency for improvement compared with those during the placebo period or the baseline. Limitations in the present study include statistical power. The power analysis was naturally performed for the primary endpoint and not the secondary endpoints. It is therefore possible that some of the positive trends in the secondary outcome measures may have been statistically significant with a larger sample size. The frequencies of cataplexy and sleep paralysis were found to be very low. The patients would have been successfully treated for these symptoms. In order to properly assess the effect on cataplexy and sleep paralysis, it would be effective to restrict concomitant medications for these symptoms. However, in conducting intervention studies that may make symptoms worse, the safety and ethics must be fully taken into consideration.

SNP rs5770917 adjacent to the gene encoding CPT1B has been found to be associated with narcolepsy [16]. CPT1B is a rate-limiting enzyme in β-oxidation of long-chain fatty acids. The expression levels were also associated with SNP rs5770917 genotype [16], [17]. In this clinical trial, no significant association was observed between SNP rs5770917 and the primary endpoint. In addition, serum levels of total carnitine, free carnitine, acylcarnitine and triglycerides which were improved by l-carnitine were not significantly associated with SNP rs5770917. These results suggest that it may not be necessary to limit narcolepsy patients given l-carnitine using SNP rs5770917 genotype. However, the possibility of false negative cannot be denied because this study was not designed to assess the effect of SNP rs5770917, therefore a further study is required.

We observed that half of the study patients were obese. Average triglyceride levels during the placebo period were above the normal range, and 4 patients taking placebo showed abnormally low acylcarnitine levels (Table 2). These results reconfirmed previous studies [6], [17], suggesting that fatty acid metabolism would be associated with the pathophysiology of narcolepsy. Oral administration of l-carnitine was associated with triglyceride reduction and acylcarnitine increase, indicating that carnitine absorption and utilization are generally similar in narcolepsy patients and in normal controls. Triglyceride could be effectively utilized for fatty acid oxidation, resulting in the increase of acylcarnitine and facilitation of fatty acid oxidation. Treatment with l-carnitine could have the potential to reverse metabolic abnormalities in narcolepsy patients. However, there were no differences in BMI and total cholesterol between l-carnitine and placebo periods (Table 2). Generally, clinical trials for body weight and serum lipid profile in obese patients have been conducted for 6 months to 4 years [31]. The duration of the l-carnitine period in this study was only 8 weeks. Therefore, long term study is necessary to properly evaluate the effects of l-carnitine on obesity and lipid profile.

We set the endpoints for this study based on subjective measurements such as sleep logs, and questionnaires about daytime sleepiness (JESS) and QOL (SF-36 subscales), because our research focused on the changes in levels of sleepiness and psychological conditions in daily life of participants. Multiple sleep latency test (MSLT) is the standard test to objectively evaluate patients with excessive daytime sleepiness [32]. It would provide further information to use objective measurements such as MSLT for sleepiness in a future study. However, MSLT measures the tendency toward falling asleep (sleep propensity) in a lying position with few arousal signals from sensory input in a sleep laboratory. The outcome of MSLT measures reflect short-term “state” sleepiness and could be different from the “trait” sleepiness, the average conditions of many different situational sleep propensities that reflect the activities of daily life [33].

We tried to measure the changes of the global level of sleepiness using total dozing off time on sleep logs and JESS rating. They showed significant correlation with each other (correlation coefficient = 0.34, *P* = 0.0051), suggesting that total dozing off time on sleep logs could measure similar “trait” sleepiness as JESS. We selected total dozing off time on sleep logs as a primary outcome measure, because sleep logs are more accurate than other retrospective self-administered questionnaires including JESS. The participants can record their daily sleep status and symptoms in sleep logs before they forget. Furthermore, the total dozing off time calculated from the consecutive data can reduce the day-to-day variation. Clinical research coordinators instructed the participants how to record events in the sleep logs and confirmed that the sleep logs were correctly described.

A sleep actigraph as an objective measure is useful for determining nocturnal sleep timing and duration and can be worn for several weeks at a time [34]. We collected actigraph data in both l-carnitine and placebo periods and compared the actograms with the sleep logs, and observed that naps and dozing off time recorded in sleep logs approximately corresponded to the period of less motor activity, confirming that the participants understood and followed the instruction of clinical research coordinators well, and sleep log data could be utilized reliably for the comparison of sleepiness within the same subject. Incidentally, total motor activity, which is a principal measure of the actigraph, could be recorded in these study participants. We simply compared the total activity between l-carnitine and placebo periods, but the result showed no significant difference.

Low serum acylcarnitine levels have been observed in patients with chronic fatigue syndrome (CFS) [35]–[37], which is a clinically defined condition characterized by severe disabling fatigue and a combination of symptoms, such as musculoskeletal pain, difficulty in concentration and sleep disturbances [38]. It has been reported that l-carnitine supplementation resulted in significant improvements in fatigue severity after two months of supplementation [39]. Other results suggest that narcolepsy patients also feel consistently fatigued [40]–[42]. The SF-36 vitality subscale score of our participants was lower than that of national standards, and the score tends to be improved by l-carnitine administration, confirming that fatigue is common among narcolepsy patients (Table 2). These results also indicate that there might be a common pathological process underlying narcolepsy and CFS since both are accompanied by low serum acylcarnitine levels, a symptom that is improved by l-carnitine treatment.

In conclusion, l-carnitine is an effective and well-tolerated treatment for daytime somnolence in narcolepsy patients, but further studies with larger numbers of patients and long-term observation periods are required to confirm its efficacy and safety, and to clarify the mechanisms underlying its benefit.

## Supporting Information

Checklist S1 **CONSORT Checklist.**
(DOC)

Protocol S1 **Trial protocol.**
(DOC)
