# Supplementary material for: Effects of Oral L-Carnitine Administration in Narcolepsy Patients: A Randomized, Double-Blind, Cross-Over and Placebo-Controlled Trial
Source: PLoS One. 2013 Jan 17;8(1):e53707. doi: 10.1371/journal.pone.0053707 (PMC3547955; doi:10.1371/journal.pone.0053707)
Supplement: Protocol S1 — Trial protocol. (DOC) [file pone.0053707.s002.doc]

Clinical Evaluation of the Effects of L-Carnitine on Narcolepsy with Cataplexy

Protocol

Protocol no. CAR01

Date Prepared: May 28, 2010

Version no. 01-007

**1. Administrative Structure**

**Medical Institutions**

| Medical institution | Address and phone number | Investigators |
| --- | --- | --- |
| Yoyogi Sleep Clinic affiliated to Neuropsychiatric Research Institute  (Currently, Yoyogi Sleep Disorder Center) | TS building, 1-24-10 Yoyogi, Shibuya-ku, Tokyo  Postal code: 151-0053  Dial number: 03-3374-9112 | Makoto Honda  Yuichi Inoue |

**2. Background**

This clinical evaluation was planned following the findings of a genome-wide analysis on narcolepsy, expression analysis of *CPT1B*, and measurement of plasma levels of acylcarnitine discussed below. The Department of Human Genetics of the University of Tokyo and others conducted genome-wide analysis of 500,000 single nucleotide polymorphisms (SNPs) to identify genetic factors related to narcolepsy. SNP rs5770917, located near the *CPT1B* gene on chromosome 22 (22q13.33), was found to be closely associated with narcolepsy. This analysis, conducted with the genomes of Japanese individuals, revealed the risk of narcolepsy to be 1.8-fold higher in those with the risk allele in SNP rs5770917, as compared to those without. SNP rs5770917 has also been associated with narcolepsy in South Koreans. Subsequently we conducted an analysis to determine whether SNP rs5770917 functionally affects mRNA level of *CPT1B* expression and found that people with the risk allele express less *CPT1B* than those without it.

Carnitine palmitoyltransferase 1b (CPT1B) is a rate-limiting enzyme involved in beta oxidation, which is the primary oxidation pathway of fatty acids. CPT1B is responsible for combining carnitine with acyl-CoA produced from fatty acids and producing acylcarnitine. Fatty acids, in the form of acylcarnitine, pass through the inner membrane of mitochondria to enter the matrix. Beta oxidation and the carnitine system have previously been reported to contribute to sleep regulation. Thus the decreased expression of *CPT1B* attributable to the above SNP rs5770917 may impact fatty acid metabolism and sleep regulation. Acylcarnitine levels in the plasma of narcolepsy patients and healthy individuals were measured and compared to determine whether narcolepsy patients may have abnormal fatty acid metabolism. Significantly more narcolepsy patients had abnormally low levels of acylcarnitine. This finding convinced us that L-carnitine, which normalizes acylcarnitine levels and promotes fatty acid metabolism, could have therapeutic value in narcolepsy.

L-carnitine is found in the body and contributes to fatty acid catabolism. L-carnitine was originally used to promote the production of energy from fatty acids in the 1980s in Western countries, and now the compound is widely used in foods or as dietary supplements and functional foods. After evaluating the safety and other aspects of L-carnitine, the Japanese Ministry of Health, Labour and Welfare approved the use of the compound in food under the Food Sanitation Law in 2002. Transient mild diarrhea has been reported at doses of 15 g, but this is attributable to changes in osmotic pressure in the intestine. No substantial adverse reactions are anticipated in this clinical evaluation.

L-carnitine thus has the potential to be a highly safe and clinically beneficial treatment. The clinical evaluation described in this protocol will be conducted as a double-blind, crossover study to verify the efficacy of 510 mg of L-carnitine in narcolepsy patients and thereby generate evidence substantiating this treatment in narcolepsy patients.

**3. Objective and Endpoints**

**Objective**

The objective is to verify the efficacy of 8 weeks of treatment with 510 mg of L-carnitine in narcolepsy patients based on sleep logs, Epworth sleepiness scale (ESS) scores, questionnaire scores including subscales of the Medical Outcomes Study 36-Item Short-Form Health Survey (SF-36) (for subjectively evaluating symptoms and overall health (physical condition)), actigraphy and BMI.

**Endpoints**

Comparison of sleep logs, ESS scores, questionnaire scores (for subjectively evaluating symptoms and overall health (physical condition)), actigraphy, BMI and blood chemistry test data (including plasma carnitine levels) determined following treatment with 510 mg of L-carnitine versus placebo (see 5.5 Endpoints for details).

**4. Subjects**

**4.1 Subject Population**

Patients having narcolepsy with cataplexy (hereafter, “narcolepsy”)

**4.2 Inclusion Criteria**

Narcolepsy patients satisfying all of the following criteria will be enrolled:

• Male or female at least 15 years of age (at time of informed consent)

• Outpatient

• Has personally given written informed consent

**4.3 Exclusion Criteria**

Patients to whom any of the following criteria applies will not be enrolled:

• History of epileptic seizures

• Currently uses acenocoumarol (an anticoagulant)

• Has hypersensitivity to L-carnitine or any placebo ingredient

• Is or may be pregnant, is breastfeeding, or wishes to become pregnant during the clinical evaluation

• Is participating in another clinical study

• Is found by an investigator to be ineligible as a subject

**4.4 Target Sample Size**

Target sample size: A full analysis set of 30.

*Definition of full analysis set:*

The full analysis set is the population consisting of all assigned subjects except for those who do not receive L-carnitine or placebo at all

**5. Methods**

**5.1 Design**

Double-blind, crossover study

510 mg of L-carnitine

Informed consent

Placebo

510 mg of L-carnitine

Placebo

Visit 1　　　　　Visit2　　　　　　Visit3　　　　　Visit4　　　　　　Visit5

Week 0 Week 4 Week 8 Week 12 Week 16

Treatment with L-carnitine or placebo

**5.2 Schedule**

|  | Step 1: 8 weeks  (L-carnitine or placebo) | | |  | Step 2: 8 weeks  (L-carnitine or placebo) | | |
| --- | --- | --- | --- | --- | --- | --- | --- |
|  | Visit1 | Visit2 | Visit3 | | | Visit4 | Visit5 |
|  | Week 0 | Week 4 | Week 8 | | | Week 12 | Week 16 or on discontinuation |
|  |  | Visit 1 + 4 weeks ± 14 days | Visit 2 + 4 weeks ± 14 days | | | Visit 3 + 4 weeks ± 14 days | Visit 4 + 4 weeks ± 14 days |
| Informed consent | ○ |  |  | | |  |  |
| Symptoms and signs | ○ | ○ | ○ | | | ○ | ○ |
| Physical examination | ○ | △ | ○ | | | △ | ○ |
| ESS and other  questionnaires | ○ |  | ○ | | |  | ○ |
| Laboratory tests  (blood sampling) |  | △ | ○ | | | △ | ○ |
| Adverse event  assessment |  |  |  | | |  |  |
| Actigraphy |  |  |  | | |  |  |
| Sleep logs |  |  |  | | |  |  |

○: Mandatory, △: Perform if deemed necessary; Physical tests: Determine blood pressure, pulse rate, height, weight, and body mass index (BMI)

Height need be measured only once during the clinical evaluation. Height determined at another time may be used.

Duration of L-carnitine and placebo treatment will not exceed 11 weeks.

**5.3 Duration of Clinical Evaluation**

May 2010 to December 2010 (final enrollment deadline: July 2010)

**5.4 Restrictions**

## Prohibited concomitant drugs

Acenocoumarol (an anticoagulant)

Other investigational products

**5.5 Endpoints**

Sleep logs: Total time for dozing off during the daytime (primary endpoint), the number of naps during the daytime, the number of sleepiness episodes during the daytime, the number of cataplexy attacks, and the number of sleep paralysis episodes in the subjects receiving L-carnitine will be compared against those in subjects receiving placebo. Data obtained during the first 2 weeks (including the first day of treatment) will be excluded because of potential carry-over effect. Nonetheless subjects will be asked to keep their sleep logs during this period.

ESS and questionnaire: The ESS scores and subject-completed questionnaires (including SF-36 subscales) will be obtained on the final day of L-carnitine or placebo treatment period and be compared.

Actigraphy: The activity levels of the subjects will be measured with an actigraph and the activity levels of L-carnitine and placebo treatment periods will be compared. Activity levels will be measured from 11:59 p.m. on the 4th day of Visits 2 and 4 (e.g., Tuesday of the following week if the visit is on Friday) to 7:00 a.m. on the 16th day of the visit (e.g., Sunday of the week after the next if the visit is on Friday).

BMI changes in subjects receiving placebo will be compared against those receiving L-carnitine. Similarly, blood chemistry test data (total cholesterol, triglycerides, and carnitine fraction) of subjects receiving placebo will be compared against the data of subjects receiving L-carnitine.

**5.6 Laboratory Tests**

Hematology and blood chemistry tests will be performed on the days shown in the schedule (Visits 3 and 5).

| Category | Test parameters |
| --- | --- |
| Hematology tests | Red blood cell count, hemoglobin, hematocrit, platelet count, white blood cell count, mean corpuscular volume, mean corpuscular hemoglobin, mean corpuscular hemoglobin concentration |
| Blood chemistry tests | Bilirubin (total and direct), ALT (GPT), AST (GOT), γ-GTP, total cholesterol, triglycerides, creatinine, Na, K, Cl |

Sampling volume: 25 mL per testing session

Other than the above-mentioned test parameters, blood samples can be retained and used for genotyping (primarily SNP rs5770917) and analyzing the carnitine fraction and acylcarnitine fractions under subject consent.

**5.7 Physical Examinations**

Sitting blood pressure (systolic and diastolic), sitting pulse rate, height, weight, and body mass index (BMI) will be determined on the days shown in the schedule (Visits 1, 3, and 5). Height needs to be measured only once during the clinical evaluation. Height determined at another time may be used.

**5.8 Discontinuation Criteria**

Appropriate countermeasures will be taken for the affected subject and this clinical study will be discontinued in the event of any of the following:

1. An exclusion criterion is found to be applicable after the start of the clinical evaluation.
2. The investigator determines that an adverse event makes it infeasible to continue the clinical evaluation.
3. A subject or legally acceptable representative requests discontinuation.
4. A subject is found to be pregnant.
5. Worsening of the underlying disease or primary disease makes it infeasible to continue the clinical evaluation.
6. A subject is transferred to another hospital or is for another reason unable to visit.

The investigator will contact any subject who, without approval, stops visiting by telephone or other means, to ask if the subject is healthy and to urge the subject to visit.

**6. About L-carnitine and Placebo**

**6.1 Description, Handling, and Storage**

The 170 mg of L-carnitine and placebo used in the clinical evaluation are formulated as capsules.

| Reason for use |  | Ingredient and strength (check ingredients, particularly in the capsules) |
| --- | --- | --- |
| Study ingredient | 170 mg of L-carnitine | One capsule contains 170 mg of L-carnitine, a white, odorless substance. |
| Control ingredient | Placebo | One capsule contains about 150 mg of glucose, a white, odorless substance. |

L-carnitine and placebo will be stored at room temperature away from high-temperature, high-humidity locations and away from direct sunlight.

**6.2 Dosage and Administration**

Capsules containing 510 mg of L-carnitine or the placebo will be used. The subjects will take 3 capsules per day (2 in the morning and 1 in the evening).

**6.3 Assignment**

The assigner will randomly assign each subject in the place without anybody except for the assignment assistant. Taku Miyagawa of the Graduate School of Medicine, University of Tokyo will serve as the assigner. The assigner will prepare a key code table and retain this table from the start of assignment until key open. If a particular assignment must be known for a subject before unblinding (e.g., if a serious adverse event occurs and the investigator decides that the assignment must be known to ensure safety), the assignment for the affected subject in the key code table may be identified, and may be revealed to the subject.

**6.4 Packaging and Labeling**

Capsules of L-carnitine and placebo are available in different plastic bottles. As shown in the example below, the products will be controlled for the subjects in numerical order. The subject number and “A” are printed on the plastic bottle used in the first period (step 1). Similarly, the subject number and “B” are printed on the plastic bottle used in the second period (step 2).

Example: Subject 1: 1-A and 1-B

Subject 2: 2-A and 2-B

…

Subject 20: 20-A and 20-B

…

Subject 30: 30-A and 30-B

**6.5 Dispensing Procedures**

One plastic bottle will be used in each step, and the bottle for a given step will be handed to the subject on the first day of the treatment period. Each bottle contains 231 capsules (which is enough for 11 weeks). Following treatment, each subject will return the remaining L-carnitine or placebo together with the plastic bottle. The dates of dispensing and return of the bottle will be entered in the capsule dispensing chart.

**7. Adverse Events**

**7.1 Adverse Events**

An adverse event is defined as any untoward medical occurrence in a patient or subject who takes L-carnitine or placebo, which is not necessarily causally related to L-carnitine or placebo.

More specifically, an adverse event is any untoward and unintended sign, symptom, or (newly occurring or worsening) disease that occurs following treatment with L-carnitine or placebo.

*Examples that constitute an adverse event*

- Marked or unexpected worsening or deterioration in a studied symptom/indication
- Chronic or ongoing deterioration in a symptom present before the start of the clinical evaluation (including increases in symptom frequency or severity)
- A symptom, newly identified or diagnosed after the start of treatment, that may have been present before the start of the clinical evaluation
- A sign, symptom or sequela for which interaction is suspected
- A sign, symptom or sequela for which L-carnitine or placebo overdose is suspected

*Examples that do not constitute an adverse event*

- A medical or surgical procedure (e.g., endoscopy, appendectomy)
  (although any symptom necessitating such procedures is an adverse event)
- When no medically untoward event occurs (e.g., admission for social reasons or convenience)
- A disease or symptom which is present before the start of the clinical evaluation worsens within an expected range or does not worsen
- When the studied disease or its associated signs or symptoms do not worsen beyond the range estimated from the condition of the subject

**7.2 Adverse Event Observation Period, Frequency, and Methods**

Information about adverse events (including serious adverse events) will be collected while the procedures specified in the protocol are executed.

The investigators will interview the subjects about adverse events on the days of visits (or contact) during the steps.

The investigators will identify adverse events with questions such as:

• “Has anything changed physically since your last visit (day of contact)?”

• “Have you begun taking a new drug since your last visit (day of contact)?”

**7.3 Assessing Causality**

The investigators will assess the causal relationship between each adverse event (including serious one) and L-carnitine. The investigators will assess causality on the two-grade scale shown below. “Is there a reasonable possibility that L-carnitine treatment caused the event?”

• Yes

• No

Causality assessments will be reevaluated and, as necessary, revised if new information is obtained.

**7.4 Follow-up for Adverse Events**

Each investigator will perform as much follow up as possible for each adverse event (including serious one), collecting additional information about the condition of the subject. The investigators will perform any necessary additional tests to elucidate the nature and/or causality of adverse events.

**7.5 Definitions of Serious Adverse Events**

A serious adverse event is an adverse event that:

1) Results in death

2) Results in persistent or marked disability/incapacity

3) Is life threatening

4) Requires hospitalization or prolongation of hospitalization for treatment

5) Is another medically notable condition ^1^

6) Is a congenital anomaly

1 In general, hospitalization refers to the holding of a subject in a hospital or emergency care facility (normally for at least one night) to undergo observations or treatment that cannot be appropriately performed in an examination room or on an outpatient basis. Prolongation of hospitalization for a concurrent disease and a concurrent disease satisfying the definition of another serious adverse event also constitute adverse events. Hospitalization for an elective procedure for an existing symptom that has not worsened since the start of the clinical evaluation does not constitute an adverse event.

**8. Handling of Personal Information and Results**

**8.1 Protection of Personal Information**Data and information (other than subject medical records) will be maintained in confidentiality. To protect personal information, all provided samples and information on sleep will be stripped of the address, name, phone number, and other personally identifiable information and instead indicated with a numerical code, so that the individual cannot be identified.

**8.2 Publication of Results**Results may be presented at a conference or published in a log. All personally identifiable information will be removed prior to publication.

**8.3 Ownership of Intellectual Property Derived from Clinical Evaluation**Any intellectual property derived through clinical evaluation will be the property of the study centers and personnel, and not the property of the subjects.

**9. Ethics**

**9.1 Informed Consent**

Before beginning the clinical evaluation, the investigator will fully inform the candidate subject about the clinical evaluation using the written information and informed consent form approved by the ethics committee. The investigator will provide each candidate subject with an opportunity to ask questions and sufficient time to decide whether to participate. After a subject has provided informed consent, the subject’s signature or personal seal and date of consent will be added to the informed consent form. The informing party will also enter the day of the explanation on and sign or seal the informed consent form. If a clinical research associate provides a supplementary explanation, that person will also enter the day of the explanation on and sign or seal the informed consent form.
